# Pure Laparoscopic Versus Open Liver Resection for Primary Liver Carcinoma in Elderly Patients: A Single-Center, Case-Matched Study

**DOI:** 10.1097/MD.0000000000001854

**Published:** 2015-10-30

**Authors:** Xi-Tao Wang, Hong-Guang Wang, Wei-Dong Duan, Cong-Ying Wu, Ming-Yi Chen, Hao Li, Xin Huang, Fu-Bo Zhang, Jia-Hong Dong

**Affiliations:** From the Department of Hepatobiliary Surgery (X-TW, H-GW, W-DD, M-YC, HL, XH, F-B, J-HD), Chinese PLA General Hospital, Beijing; School of Medicine (X-TW, XH, F-B, J-HD), Nankai University, Tianjin; and Institute of Systems Biomedicine (C-YW), Peking University, Beijing, China.

## Abstract

Pure laparoscopic liver resection (PLLR) has been reported to be as safe and effective as open liver resection (OLR) for liver lesions, and it is associated with less intraoperative blood loss, shorter hospital stay, and lower complication rate. However, studies comparing PLLR with OLR in elderly patients were limited. The aim of this study was to analyze the short-term outcome of PLLR versus OLR for primary liver carcinoma (PLC) in elderly patients.

Between January 2008 and October 2014, 30 consecutive elderly patients (≥70 years) who underwent PLLR for PLC were included into analysis. Sixty patients who received OLR for PLC during the same study period were also included as a case-matched control group. Patients were well matched in terms of age, sex, comorbid illness, Child Pugh class, American Society of Anesthesiologists grade, tumor size, tumor location, and extent of hepatectomy.

No significant differences were observed with regard to patient preoperative baseline status, median tumor size (Group PLLR 4.0 cm vs Group OLR 5.0 cm, *P* = 0.125), tumor location, extent of hepatectomy, and operation time (Group PLLR 133 minutes vs Group OLR 170 minutes, *P* = 0.073). Compared with OLR, the PLLR group displayed a significantly less frequent Pringle maneuver application (10.0% vs 70.0%, *P* < 0.001), less blood loss (100 vs 300 mL; *P* < 0.001), shorter hospital stay (5 vs 10 days; *P* < 0.001), and lower total hospitalization cost ($9147.50 vs $10,867.10, *P* = 0.008). The postoperative complication rates were similar between groups (Group PLLR 10.0% vs Group OLR 16.7%; *P* = 0.532). There was no hospital mortality in both groups.

PLLR for PLC is as safe and feasible as OLR, but with less blood loss, shorter hospital stay, and lower hospitalization cost for selected elderly patients.

## INTRODUCTION

Owing, in part, to the increasing overall life expectancy, the populations of industrialized countries are steadily aging.^1^ Meanwhile, with the rising incidence of primary liver carcinoma (PLC), the number of elderly patients who need liver resection treatment is estimated to be increasing.^2^ Surgeons are now confronted with an older patient population who has a decreased organ function reserve and a longer list of medical comorbidities. Considering the benefit-to-risk ratio for the patients with advanced age, liver resection would be quite risky.^3,4^ However, studies from different medical centers have confirmed the safety and feasibility of open liver resection (OLR) for elderly patients with an acceptably low complication rate and satisfying oncological outcomes.^5–7^

On the contrary, advances in laparoscopic techniques and instruments have rendered laparoscopic procedure more safe and feasible in surgical opertation.^8,9^ Over last decades there has been an increasing trend toward a minimally invasive approach of liver resection with less blood loss, shorter hospital stay, and without compromised complication rate or oncological outcomes comparing with open surgery in younger patients populations.^10–12^

Nonetheless, limited studies have compared the results of pure laparoscopic liver resection (PLLR) versus OLR for PLC in patients with advanced age, and it remains unclear if the elderly patients could also benefit from the minimally invasive approach of liver resection as the younger patients did.^13^ Therefore, the objective of this retrospective case-matched study was to compare the short-term results between PLLR and OLR for PLC in elderly patients based on our experience.

## MATERIALS AND METHODS

### Patient Population

This is a retrospective analysis on a prospectively collected database of all consecutive elderly patients (≥70 years), who have underwent PLLR for malignant PLC from January 2008 to October 2014 at Chinese PLA General Hospital (Beijing, China). Pure laparoscopic procedure was defined as the entire liver resection being completed through laparoscopic ports, according to the Louisville Statement 2008.^14^ Patients with conversion, combined surgical procedures, or incomplete work-ups were excluded from this analysis, and data of patient demographics, preoperative liver function, tumor features, types of resection, postoperative outcomes, and total cost were reviewed.

For comparison, the patients who received PLLR for PLC were matched with control patients who underwent conventional OLR during the same study period in a 1:2 ratio, according to age, sex, comorbid illness, Child-Pugh class, American Society of Anesthesiologists (ASA) grade, tumor size, tumor location, and extent of hepatectomy. The Institutional Review Board of Chinese PLA General Hospital approved this study. As an observational study, this analysis followed the guidance of STROBE statement.^15^

### Preoperative Evaluation

A complete blood and liver function test as well as routine cardiorespiratory evaluation through electrocardiogram and spirometry were implemented for preoperative assessment. For patients with cardiac comorbidities or high index of suspicion of occult cardiopulmonary disease, an echocardiogram and lung function test were also performed. Exclusion criteria for liver resection were congestive heart failure, high-risk coronary artery disease, recent stroke, and chronic obstructive pulmonary disease that significantly limited moderate exertion. For cirrhosis patients, liver function was assessed by routine liver biochemistry and indocyanine green clearance test. Only patients with Child-Pugh class A and indocyanine green retention rate <14% at 15 minutes after injection were considered to be offered liver resection. With respect to the tumor, resectability was defined by the absence of extrahepatic metastasis and tumor thrombus in the main portal vein and inferior vena cava on triple-phase computed tomography or magnetic resonance imaging. Laparoscopic approach would be considered based on location of lesions, tumors’ proximity to major vascular structures, and extent of resection. Lesions that located in posterior segments (Couinaud segments 1, 7, and 8), and had intimate contact with major hepatic vessel structures or that required a locoregional lymph node dissection, were excluded from laparoscopic surgery because it was difficult to obtain an R0 liver resection for these patients.

### Surgical Procedure

The extent of hepatectomy was recorded according to the Brisbane 2000 terminology of liver anatomy and resections.^16^ Patients with small and peripherally located tumors would receive wedge resection. Anatomical resection would be performed if preoperative indocyanine green test showed that the patient's liver function could tolerate it. A 1-cm gross margin was aimed for during the liver resection in both groups. Margin status was defined as R0 for microscopically negative for tumor invasiveness or as R1 for macroscopically negative but microscopically positive for tumor invasiveness.

For the PLLR approach, detailed information about surgical technique routinely used in our department has been described in previous reports.^17–19^ In brief, the patient was usually placed in a 30° reverse Trendelenburg position with legs spread apart (French position) and tilted 30° to the left or right according to the lesion location. The primary surgeon stood between the legs with one assistant on each side. The pneumoperitoneum was maintained at 12 mm Hg to minimize the risk of gas embolism. Four to 5 working ports sized between 5 and 12 mm were used in a position similar to that used in the 4-hole laparoscopic cholecystectomy, and appropriate adjustments were made based on tumor location. After a standard diagnostic and staging laparoscopy, intraoperative laparoscopic ultrasonography was performed in every patient to confirm tumor numbers, lesion position in relation to main intrahepatic vascular and safety resection margin. Liver parenchymal transection was performed using a combination of harmonic scalpel, ultrasonic dissector, and bipolar forceps. Small vessels were coagulated directly, and large vessels (diameter ≥3 mm) were occluded using titanium clip or Hem-o-lok clamping. Portal pedicles and major hepatic veins were divided by application of vascular stapling devices. During liver transection, the intravenous fluid was carefully controlled. Central venous pressure was maintained at a low level (<5 mm Hg), and Pringle maneuver was used if necessary. The resected specimens were placed in a plastic retrieval bag and removed through a 5-cm horizontal suprapubic (Pfannenstiel) incision, which was immediately closed with subcuticular suture. Abdominal drainage was usually omitted.

For the OLR approach, the liver resections were performed through a right subcostal incision, which in some cases was extended to the midline. After exploration of the abdominal cavity, intraoperative ultrasonography was performed routinely to ascertain the tumor location and to exclude additional tumors in the liver remnant. The portal triad was systematically dissected to enable performance of the Pringle maneuver when needed. Parenchymal transection was achieved with the harmonic scalpel and high frequency electrocauterization. Control of minor bleeding was obtained with monopolar electrocoagulation. Clips or nonabsorbale sutures were used for the ligation of major vessels.

All resections were performed with curative intent by the same team of surgeons specialized in hepatobiliary surgery. All intraoperative parameters, including type and duration of vascular clamping, surgery duration, blood loss, and subsequent blood transfusion, were recorded. Patients with any of the medical risk factors would be transferred to the intensive care unit (ICU) after operation and then transferred to the general ward when the condition became stable.

### Statistical Analysis

Descriptive statistics were expressed as median with range for continuous variables and as number with percentage for categorical data. Mann-Whitney *U* test was used for comparing continuous variables. Pearson χ^2^ test or Fisher exact test, if appropriate, was used for comparing categorical variables. A probability (*P*) value <0.05 was considered statistically significant. Statistical analysis was performed using SPSS, version 20.0 for Windows (IBM Corporation, Armonk, NY).

## RESULTS

**FIGURE 1:**
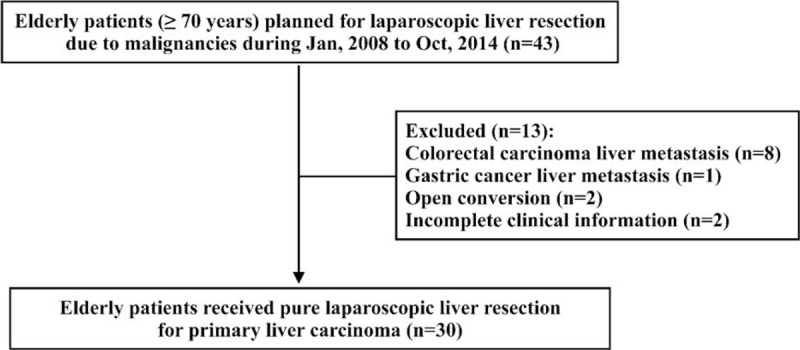
Flow chart showing the patient selection process.

From January 2008 to October 2014, there were 43 consecutive elderly patients (≥70 years) planning to receive laparoscopic liver resection for liver malignancies at our department. Among the 43 cases, 13 patients were excluded from this analysis for colorectal cancer liver metastasis (n = 8), gastric cancer liver metastasis (n = 1), open conversion (n = 2), and incomplete clinical information (n = 2) (see Fig. 1). Of the 2 conversion patients, 1 had a large lesion (>05 cm) located at the left lateral section and the tumor was severely adhesive to the greater omentum, and the other required conversion because of the middle hepatic vein bleeding during parenchymal transection for a segment 5 tumor. Eventually, a total of 30 patients with PLLR for PLC were included in this study, and they were well matched with 60 patients who received OLR for PLC during the same study period. The preoperative patient characteristics were shown in Table 1. Comparing the 2 groups, there were no difference with regard to age, sex, smoking or alcohol history, body mass index, upper abdominal operation history, comorbid illness, ASA grade, and alpha-fetoprotein level. Patients in both groups had comparable liver function in terms of Child-Pugh classification, serum alanine aminotransferase (ALT), aspartate aminotransferase (AST), prothrombin time, international normalized ratio, albumin, and total bilirubin level (see Table 1). Hepatitis B virus (HBV)/hepatitis C virus (HCV) infection patients were similarly distributed in the 2 groups (Group PLLR 36.7% vs Group OLR 56.7% for HBV, *P* = 0.117; Group PLLR 13.3% vs Group OLR 13.3% for HCV, *P* = 1.000; see Table 1). According to the final pathology, 46.7% of patients in PLLR group had liver cirrhosis and the proportion in OLR group was 40.0% (*P* = 0.652; see Table 1). The median lesion size was also comparable between the 2 groups (Group PLLR 4.0 cm vs Group OLR 5.0 cm, *P* = 0.125). Solitary lesion was developed in 90.0% of the patients in PLLR group and 88.3% of the patients in OLR group (*P* = 1.000). In the final pathological findings, the 2 groups had similar tumor type distribution (Group PLLR 83.3% vs Group OLR 95% diagnosed as hepatocellular carcinoma, and Group PLLR 16.7% vs Group OLR 5.0% diagnosed as cholangiocarcinoma, respectively; *P* = 0.111; see Table 2), and there was no difference in Edmondson grading of tumor between the 2 groups (*P* = 0.947).^20^

**TABLE 1:**
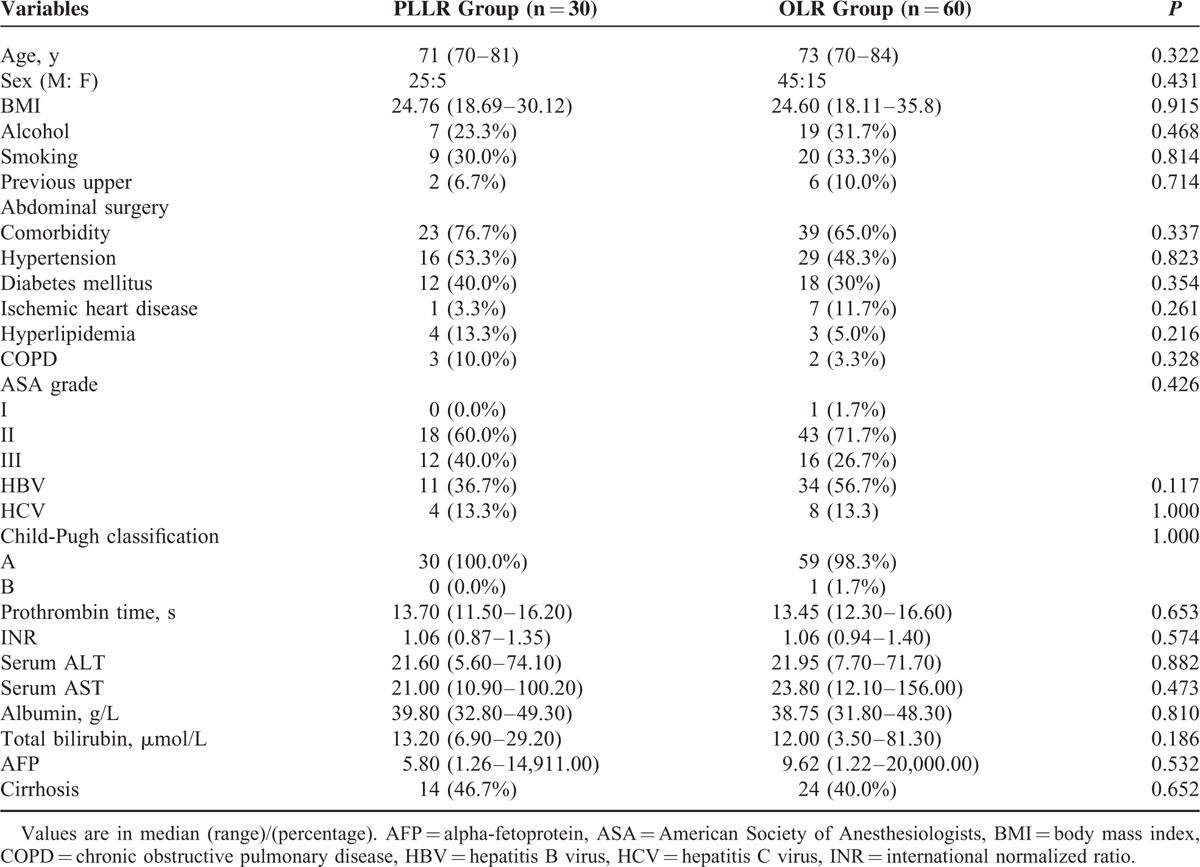
Comparison of Preoperative Characteristics of the 2 Groups

**TABLE 2:**
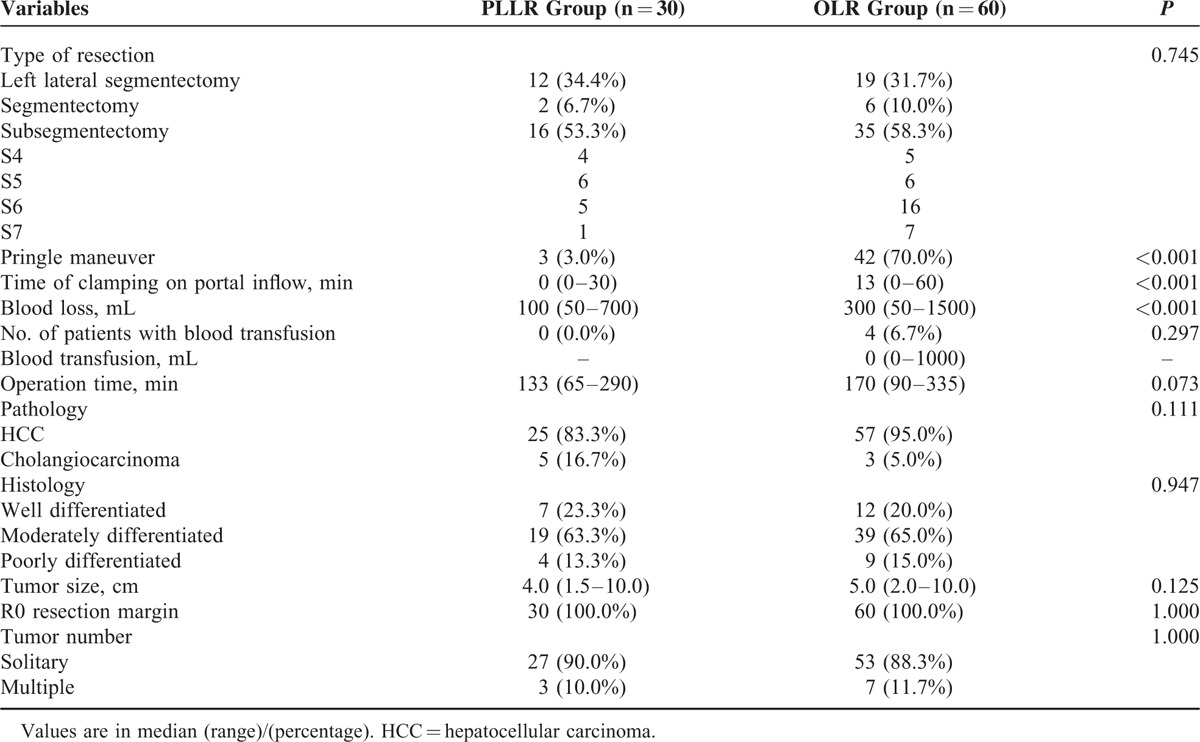
Comparison of Surgical Procedures and Results of the 2 Groups

The operative details were shown in Table 2. The liver resection types of these 2 groups were similar (*P* = 0.745), and no significant difference was observed in the operation time (133 minutes for PLLR vs 170 minutes for OLR, respectively; *P* = 0.073). The median blood loss in PLLR group was 100 mL (range, 50–700 mL), whereas it was 300 mL (range, 50–1500 mL) in the OLR group (*P* < 0.001). Four patients in OLR group required blood transfusion while no patient in PLLR group needed it (*P* = 0.297). There was a significant difference in the use of Pringle maneuver between the 2 groups (3 in the PLLR group vs 42 in the OLR group, *P* < 0.001), and the median time of clamping on portal inflow was 0 and 13 minutes in PLLR group and OLR group, respectively (*P* < 0.001). R0 resection was achieved in all patients.

**FIGURE 2:**
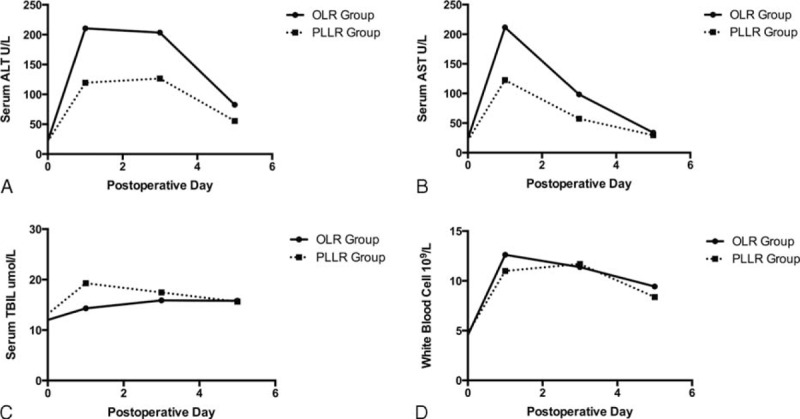
Postoperative changes in alanine aminotransferase, aspartate aminotransferase, total bilirubin levels, and white blood cell counts: (A) alanine aminotransferase level (U/L); (B) aspartate minotransferase level (U/L); (C) total bilirubin level (μmol/L); and (D) white blood cell (10^9^/L).

Postoperative data were shown in Table 3. After operation, 3 patients of PLLR group were transferred into ICU, whereas 23 patients in OLR group were transferred (*P* = 0.006). The rates of postoperative recovery between the 2 groups were also significantly different in terms of postoperative first pure liquid diet (0.98 days [range, 0.44–3.69 days] in PLLR group vs 1.62 days [range, 0.55–6.58 days] in OLR group, *P* = 0.017), first mobilization out of bed (1.74 days [range, 0.60–3.75 days] in PLLR group vs 2.78 days [range, 0.61–9.87 days] in OLR group, *P* < 0.001), and length of hospital stay (LOS) (5 days [range, 3–16 days] in PLLR group vs 10 days [7–22 days] in OLR group, *P* < 0.001). Serum ALT, AST levels, and white blood cell counts peaked on postoperative day 1 and trended to be normalized on postoperative day 3 in both groups (see Fig. 2). But median serum ALT and AST levels were significantly lower in the PLLR group than the OLR group during the first 3 days after operation. Although serum total bilirubin levels also elevated postoperatively, it remained within the normal ranges in the 2 groups. The morbidity rate was 10.0% (3 cases) in the PLLR group and 16.7% (10 cases) in the OLR group (*P* = 0.532). Even though there were 2 cases with Clavien-Dindo Type IVa complication in the OLR group (one developed heart failure and the other developed renal failure; with proper supportive treatment in the ICU, they finally discharged on postoperative day 12 and postoperative day 22, respectively),^21^ no difference was detected in the distribution of the complication types (see Table 3). There was no hospital mortality in both groups. As for the overall cost, a significant difference existed, with a median expense of $9147.50 (range, $3736.81–$26,808.14) versus $10,867.10 (range, $4854.89–$20,366.78) in the PLLR and OLR groups, respectively (*P* = 0.008).

**TABLE 3:**
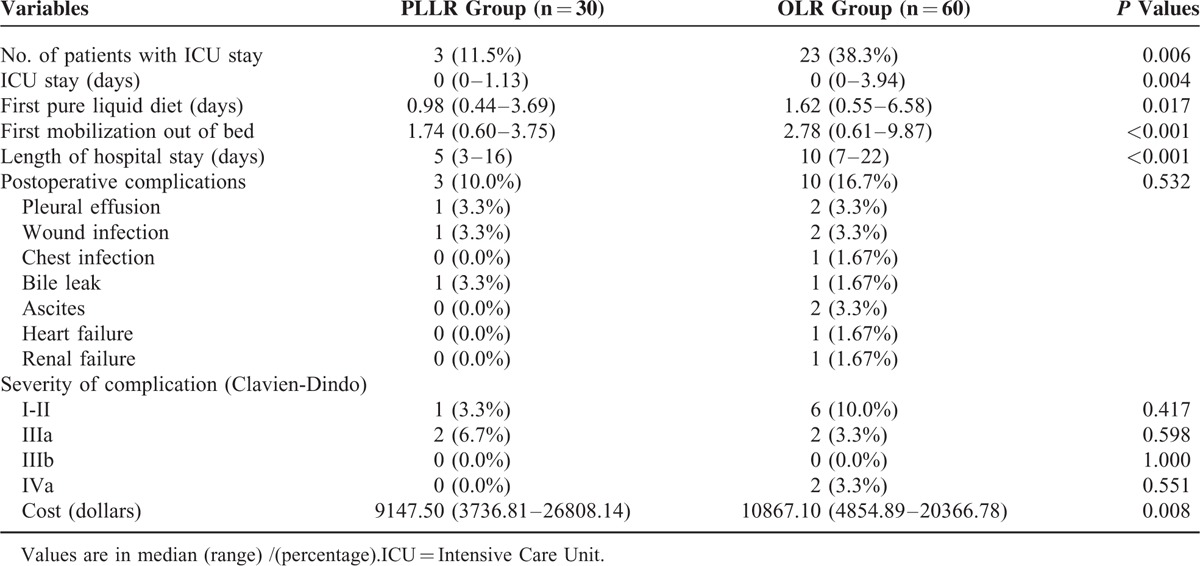
Comparison of Postoperative Outcomes of the 2 Groups

## DISCUSSION

As our populations advance in age, people with primary liver malignant neoplasm is expected to be largely increased, leading to a growing need for surgical resection.^1,2^ Owing to comorbidities, underlying liver diseases, and advanced age, liver resection for elderly patients remains challenging.^3,4^ Nevertheless, previous reports have shown successful open hepatic resections performed on elderly patients.^6,22–24^ With the improvements of laparoscopic instruments and the accumulation of surgery experience, laparoscopic liver resection as an alternative to open surgery is gaining increasing popularity because of significantly lower blood loss, blood transfusion requirements, and shorter LOS.^25–27^ Nonetheless, it remains uncertain if the benefit of laparoscopic approach could also be attained in elderly patients. Our study was engaged to evaluate the impact of the pure laparoscopic approach on short-term perioperative outcomes in elderly patients with PLC. To our best knowledge, our case-matched study is the largest collection reported to date that compared the outcome of PLLR with the conventional open approach in elderly patients with PLC.

Liver resection has been associated with increased blood loss and blood product transfusion when compared with other surgical procedures, leading to increased risk of short-term or long-term morbidity and mortality.^28,29^ In accordance with other reports,^10,13,30^ the blood loss in our study was significantly decreased in PLLR group (Group PLLR 100 mL vs Group OLR 300 mL, *P* *<* 0.001; see Table 2). No patient in the PLLR group required blood transfusion, though no significant difference was observed when compared with OLR group (Group PLLR 0 vs Group OLR 4, *P* = 0.297; see Table 2). Several factors may contribute to the decreased blood loss in the laparoscopic group. First, major resection (≥3 Couinaud segmnets) was rare (only 1 patient received left hemihepatectomy in the PLLR group), which may have significant effects in reducing severe venous bleeding risks. Second, the application of modern high-definition laparoscopy allows more meticulous hemostasis, which offered the surgeon a very clear view with magnification. Third, the raised intra-abdominal pressure from the pneumoperitoneum may result in a relative reduction in venous pressure to minimal oozing of blood during operation. Besides much smaller surgical wall wounds in PLLR group may also decrease the blood loss. Although the Pringle maneuver was an effective approach to control blood loss during parenchymal transection, it was used less frequently in PLLR group (Group PLLR 10% vs Group OLR 70%, *P* < 0.001; see Table 2). As several reports have demonstrated, total vascular inflow occlusion was associated with ischemia reperfusion injury, which led to impaired postoperative liver recovery.^31–33^ In both PLLR and OLR groups, careful administration of intravenous fluid and tight maintenance of low central venous pressure were implemented to reduce intraoperative blood loss.

As various reports have revealed reduced LOS following laparoscopic procedure,^34–36^ we observed similar outcome in our series (see Table 3). Patients underwent laparoscopic liver resection showed better organ function reserve and faster postoperative rehabilitation in terms of ICU stay, first pure liquid diet, first mobilization out of bed, and laboratory test results (eg, ALT and AST level) (see Table 3 and Fig. 2). The absence of large abdominal incision, less muscle division, less operative blood loss, infrequent utilization of Pringle maneuver, and preservation of venous collateral circulation might contribute to these superior results in PLLR group,^8,28,29,37,38^ but no significant difference of postoperative complication rate existed between these 2 groups (see Table 3). With regard to the total expense of hospitalization, the median cost of PLLR group was significantly lower than that of OLR group (see Table 3). Although the sophisticated instruments (eg, stapler devices) used in laparoscopic procedure are more costly, lower invasiveness and better organ function reservation of laparoscopic liver resection might lead to reduction of the expense for ICU and hospital stay delay, especially for elderly patients with comorbid illness.^30^

Even though improved outcomes in PLLR group have been observed in our analysis, it is worthwhile to highlight that advanced age and the presence of comorbid illness are intrinsic host factors that often limit the application of surgery treatment for liver malignancies.^13,39,40^ The indications for laparoscopic liver resection are essentially identical to those for conventional open procedure requiring careful patient selection and complete assessment of liver function.^34^ In our analysis, liver cirrhosis, which was considered as a risk factor contributing to intraoperative bleeding, postoperative sepsis, and liver failure,^22,41^ was presented in almost half of the patients in PLLR group. But none of the patients’ liver function were classified as Child-Pugh B or C, and most patients received only minor liver resections (<3 Couinaud segments) for the lesions located peripherally according to the consensus.^14^

Despite the relatively limited sample size and the retrospective nature of the analysis with a prospective recording, we opted for a case-matched, control study design to decrease inherent bias, and to our best knowledge this study represents the largest collection reported to date that analyzed the short-term outcome of PLLR versus OLR for PLC in elderly patients.

In conclusion, we reported PLLR as an alternative approach for elderly PLC patients, which allows less blood loss, shorter hospital stay, and lower hospitalization cost comparing with conventional OLR. Furthermore, larger sample and prospective comparative studies are needed to confirm the efficacy and superiority of PLLR for PLC in elderly patients.
